# EDITORIAL: NAR AWARDS 2014

**DOI:** 10.1093/nar/gku1222

**Published:** 2014-12-16

For the ninth year running, NAR and Oxford Journals have provided sponsorship and awarded travel bursaries and prizes to students in recognition of their outstanding achievements. This year, sponsorship and prizes were given at 13 different meetings. A list of the awards is presented below. Our warm congratulations go to the prize winners.

**Travel Sponsorship**

***IS3NA XXI: International Round Table on Nucleosides, Nucleotides and Nucleic Acids (IRT2014)***

Poznan, Poland

24–28 August 2014

**Poster Prizes**

***CRISPR 2014***

Berlin, Germany

14–16 May 2014

**Alexander Hynes (Université Laval, Québec, Canada)**

Adaptation in CRISPR-Cas immunity is driven by defective phage

***16th Symposium on Chemistry of Nucleic Acid Components***

Cesky Krumlov, Czech Republic

08–13 June 2014

**Dieter Buyst (Ghent University, Belgium)**

Towards DNA based esterases: uncovering Hoogsteen face regulation of the pKaH of a tethered imidazole functionality by NMR and molecular dynamics

***Non-coding RNA – From Basic Mechanisms to Cancer***

Heidelberg, Germany

22–25 June 2014

**Debojyoti Chakraborty (Dresden University of Technology, Germany)**

Systematic dissection of long non-coding RNAs involved in the regulation of embryonic stem cell pluripotency

***2014 FASEB “Machines on Genes” meeting***

Snowmass, CO, USA

22–27 June 2014

**Erich Chapman (University of Colorado, CO, USA)**

Xrn1-resistant RNA structures: Wrenches in a machine.

**James Nunez (University of California – Berkeley, CA, USA)**

Cas1-Cas2 complex formation mediates spacer acquisition during CRISPR-Cas adaptive immunity.

**Aline Simon (University of Cambridge, UK)**

The yeast Ctf4 trimer acts as a protein hub at the replication fork: characterization of its interaction with the helicase/nuclease Dna2.

***10^th^ Nucleic Acids Research Forum***

London, UK

4 July 2014

**Anne Plochowietz (University of Oxford, UK)**

Characterization of single-molecule FRET in living bacteria using protected DNA FRET standards

***FASEB Science Research Conference - Post-transcriptional control of gene expression: Mechanisms of mRNA decay***

Big Sky, MT, USA

6–11 July 2014

**Florian Ebner (University of Vienna, Austria)**

Myeloid tristetraprolin deteriorates host defense upon invasive infection by compromising neutrophil function

**Anne-Laure Lécrivain (Umeå University, Sweden)**

Mechanistic and evolutionary aspects of tracrRNA:crRNA maturation in the type II CRISPR-Cas system

***ISMB 2014***

Boston, MA, USA

13–15 July 2014

**Alex Salazar (Broad Institute, Cambridge, MA, USA)**

Investigating large sequence variants in drug resistant *Mycobacterium tuberculosis*

**Sarah Keasey (United States Army Medical Research Institute of Infectious Diseases, USA)**

The road to linking genomics and proteomics of pathogenic bacteria: from binary protein complexes to interaction pathways

***FEBS/EMBO***

Paris, France

30 August–4 September 2014

**Isabel Cruz Gallardo (University of Seville, Spain)**

The binding of TIA-1 to RNA C-rich sequences is driven by its pH dependent C-terinal RRM domain

**Sumith Kumar (Indian Institute of Science, Karnataka, India)**

Characterization of a novel phase variable type IIS restriction endonuclease from *H. pylori* strain 26695

***1st biennial Latin America Student Council Symposium (LA-SCS)***

Belo Horizonte, Brazil

7 October 27 2014

**Raony Guimarães Corrêa Do Carmo Lisboa Cardenas (Laboratory of Clinical Genomics, Brazil)**

Mendel,MD: a user-friendly online program for clinical exome analysis

**Ricardo Z. N. Vêncio (Universidade de São Paulo, Brazil)**

Sifter-T: a scalable framework for phylogenomic probabilistic protein domain functional annotation

**Javier Cáceres (Universidad Andrés Bello, Chile)**

Rational discovery of new capsaicin analogues as TRPV1 activators

***RNA Biochemistry Meeting & Workshop: RNA Modification***

Bonn, Germany

9–12 October 2014

**Juliane Schwartz**

Analysis of the translational landscape in stem cells and derived neurons

**Marco Preußner (Freie Universität Berlin, Germany)**

Circadian alternative splicing of U2af26

***OTS Annual Meeting***

San Diego, CA

12–15 October 2014

**Roya Kalantari (University of Texas Southwestern Medical Center, Dallas, TX, USA)**

Building an interaction network of nuclear Argonaute 2

**Takanori Shimo (Osaka University, Japan)**

Evaluation of exon skipping activity by locked nucleic acid-based splice-switching oligonucleotides in vitro

**Janne Turunen (Karolinska Institutet, Sweden)**

Splice-switching oligonucleotides for inhibiting proinflammatory interleukin-6 signaling

**Daniel Lazar (The Scripps Research Institute, La Jolla, CA, USA)**

Aptamer-siRNA conjugates target HIV-1 infected T cells for transcriptional gene silencing

***Post-Genome (The 4th International Conference on Science and Applied Research)***

Kazan, Russia

29 October–1 November 2014

**Nickolay Alexandrovich Kulemin (Moscow Institute of Physics and Technology, Russian Institute of Physical Medicine and Laboratory of Molecular Human Genetics of Russia)**

Methods of medical human genomes annotation

**Evgenia Temlyakova**

